# Clinical role of pretreatment albumin-to-alkaline phosphatase ratio in lung cancer: a meta-analysis

**DOI:** 10.1038/s41598-024-51844-8

**Published:** 2024-01-12

**Authors:** Yanhui Yang, Yi Wang, Xiaoliang Li, Xiaoyang Xie

**Affiliations:** Department of Cardiothoracic Surgery, The First People’s Hospital of Neijiang, Neijiang, Sichuan China

**Keywords:** Biomarkers, Medical research, Cancer

## Abstract

The association between pretreatment albumin-to-alkaline phosphatase ratio (AAPR) and clinicopathological parameters and prognosis in lung cancer is unclear. The study aimed to identify the clinical role of pretreatment AAPR among lung cancer patients. Several databases were searched for relevant studies. The primary outcome and secondary outcome were long-term survival including the overall survival (OS) and progression-free survival (PFS) and clinicopathological characteristics, respectively. The hazard ratios (HRs) and relative risks (RRs) with 95% confidence intervals (CIs) were combined. A total of 11 publications involving 10,589 participants were included in this meta-analysis. The pooled results manifested that a lower pretreatment AAPR predicted poorer OS (HR = 0.65, 95% CI 0.59–0.71, *P* < 0.001) and PFS (HR = 0.68, 95% CI 0.59–0.78, *P* < 0.001). Furthermore, subgroup analysis for the OS and PFS based on the pathological type and treatment showed similar results and pretreatment AAPR was significantly associated with worse prognosis. Besides, pretreatment AAPR was significantly associated with male (RR = 1.08, 95% CI 1.03–1.13, *P* < 0.001), poor differentiation (RR = 1.33, 95% CI 1.03–1.73, *P* = 0.029), advanced T stage (RR = 1.25, 95% CI 1.03–1.52, *P* = 0.026), N stage (RR = 1.34, 95% CI 1.15–1.55, *P* < 0.001) and TNM stage (RR = 1.14, 95% CI 1.06–1.223, *P* < 0.001). Therefore, pretreatment AAPR is significantly related to prognosis and tumor stage in lung cancer and patients with a lower pretreatment AAPR are more likely to experience poor survival and advanced tumor stage.

## Introduction

Lung cancer remains the most commonly diagnosed cancer type and leading cause of tumor-related death worldwide^1,2^. Although great advances in treatment strategies including the surgical techniques and anti-tumor drugs have been achieved in the past decade, the overall survival of lung cancer patients remains unsatisfied^3^. Besides, it is still difficult to predict the prognosis of lung cancer patients accurately and formulate the most appropriate therapy strategy now. Therefore, identifying more useful and reliable indicators contributing to the survival prediction of lung cancer patients is one of the urgent problems to be solved.

Albumin, as the most abundant protein in the blood, can reflect the body’s systemic inflammatory response and basic nutritional status. The serum albumin content in cancer patients usually decreases gradually with the progression of the disease, and studies have confirmed that the decrease of serum albumin indicates poor clinical prognosis^4,5^. Serum alkaline phosphatase is a hydrolytic enzyme concentrated in the liver, bile ducts, and kidneys, and serum levels can be significantly elevated when cancer affects the bone or liver. Studies of patients with hepatocellular carcinoma have demonstrated that the ratio of serum albumin to serum alkaline phosphatase as a prognostic factor can provide additional guidance compared with a single marker^6,7^. Therefore, the novel index, albumin-to-alkaline phosphatase ratio (AAPR), is believed to serve as a valuable prognostic indicator in cancer patients.

Xie et al. included 16 eligible studies with 5716 patients and manifested that low pretreatment AAPR was related to poor prognosis in patients with cancer^8^. However, only five studies focused on lung cancer cases in their meta-analysis. Thus, whether pretreatment AAPR is a reliable prognostic indicator in lung cancer is unclear.

This meta-analysis aimed to further identify the clinical role of pretreatment AAPR based on current evidence, contributing to the accurate prediction of survival and also formulation of treatment strategy of lung cancer patients.

## Materials and methods

This meta-analysis was performed according to the Preferred Reporting Items for Systematic Review and Meta-Analyses 2020^9^.

### Literature retrieval

The PubMed, EMBASE, Web of Science and CNKI electronic databases were searched from inception to October 14, 2022. During the literature search, the following terms were used: albumin-to-alkaline phosphatase ratio, AAPR, lung, pulmonary, tumor, cancer, carcinoma, neoplasm, survival, prognosis and prognostic. In detail, the specific search strategy was as follows: (albumin-to-alkaline phosphatase ratio OR AAPR) AND (lung OR pulmonary) AND (tumor OR cancer OR carcinoma OR neoplasm) AND (survival OR prognosis OR prognostic). Meanwhile, the free texts and MeSH terms were used during the search. References cited in included studies were also reviewed for availability.

### Inclusion criteria and exclusion criteria

The inclusion criteria were as follows: (1) patients were diagnosed with primary lung cancer; (2) the serum albumin and alkaline phosphatase levels were detected before any anti-tumor treatment; (3) patients were divided into two groups according to the pretreatment AAPR and the primary outcomes including the overall survival (OS) and progression-free survival (PFS), were compared between the two groups; (4) the hazard ratios (HRs) and 95% confidence intervals (CIs) were directly provided in the articles; 5) high-quality studies with the Newcastle–Ottawa Scale (NOS) score > 5^10^.

Exclusion criteria: (1) duplicated or overlapped data; (2) letters, editorials, case reports, animal trials or reviews.

### Data collection

The following data were extracted from each included studies: the name of first author, year, country, sample size, pathologic type, treatment, tumor stage, cutoff value of AAPR, endpoint, age, gender, smoking history, differentiation degree, T stage, N stage, TNM stage, HR and 95% CI.

### Study quality assessment

The quality of all included studies was assessed according to the NOS score tool and only high-quality studies with the NOS of 6 or higher were included as above mentioned^10^.

In our meta-analysis, the literature search, selection, data extraction and quality assessment were all performed by two independent authors.

### Statistical analysis

In this meta-analysis, all statistical analyses were performed using STATA 15.0 software. Heterogeneity among studies was evaluated using I^2^ statistics and the Q-test. When obvious heterogeneity was detected, representing I^2^ > 50% and/or *P* < 0.1, the random effects model was used; otherwise, the fixed effects model was used. The HRs, relative risks (RRs) and 95% CIs were combined. Sensitivity analysis was performed to detect sources of heterogeneity and assess the stability of the pooled results. Besides, the Begg’s funnel plot and Egger’s test were performed to detect publication bias, and significant publication bias was defined as *P* < 0.05^11,12^. If we detected significant publication bias, then the nonparametric trim-and-fill method was used to re-estimate a corrective effect size after publication bias was adjusted^13^.

### Ethical statement

The authors are accountable for all aspects of the work in ensuring that questions related to the accuracy or integrity of any part of the work are appropriately investigated and resolved. All procedures performed in studies that involved human participants were in accordance with the ethical standards of the institutional and/or national research committee and with the 1964 Helsinki Declaration and its later amendments or comparable ethical standards.

## Results

### Literature search and selection

**Figure 1:**
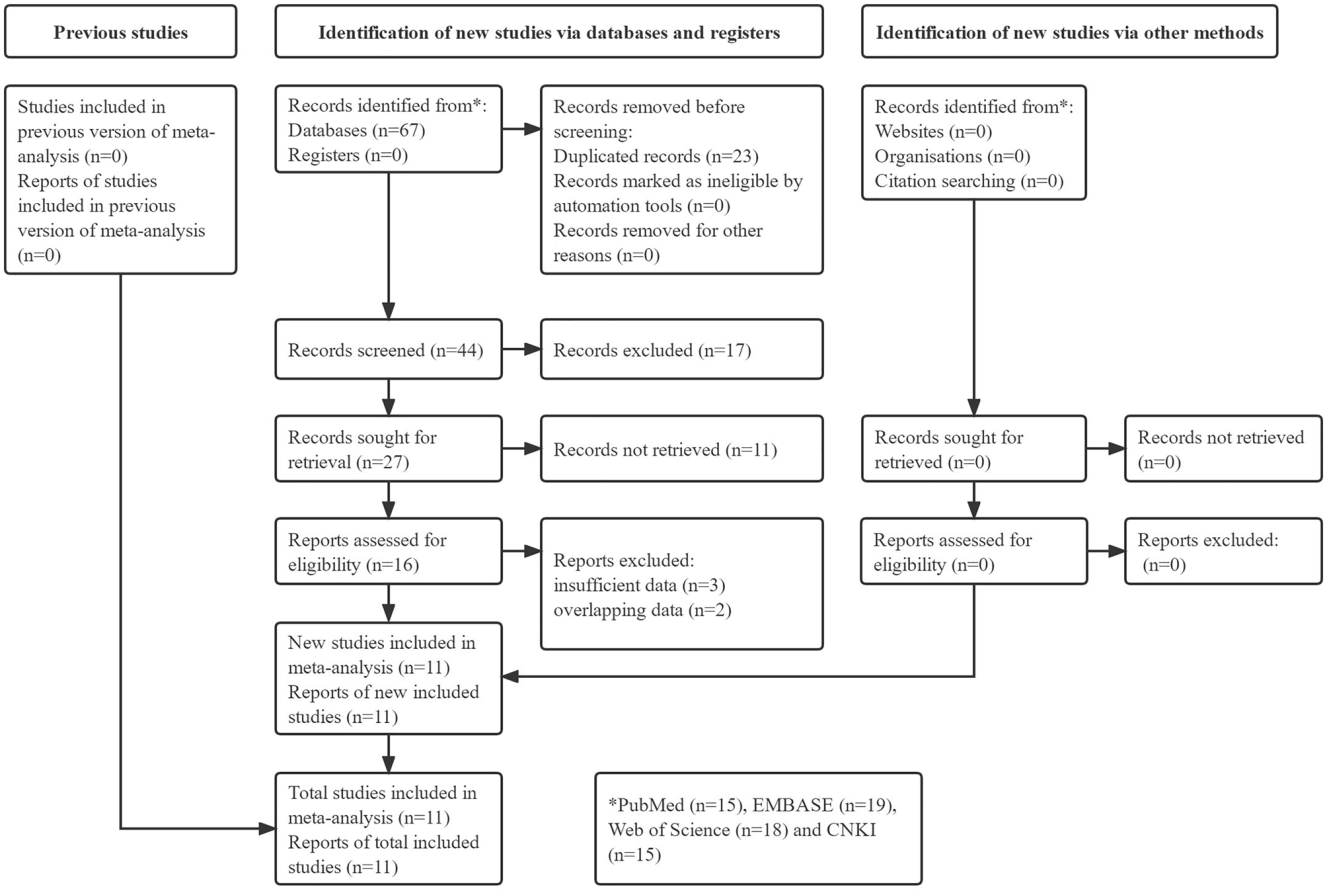
Prisma flow diagram.

Sixty-seven records were searched from the four databases initially and 23 duplicated records were removed. Then the titles and abstracts of remaining 44 records were reviewed and full texts of 16 studies were further reviewed. Finally, 11 relevant studies were included our meta-analysis^14–24^. The detailed process was displayed in the Fig. 1.

### Basic characteristics of included studies

A total of 10,589 patients were enrolled in the analysis with the sample size ranged from 116 to 7078. Except the study by Birgitte et al.^21^, all included studies were from China. Eight and four studies focused on the NSCLC and SCLC patients, respectively. The cutoff values of AAPR ranged from 0.238 to 0.64. Other detailed information was presented in Table 1.

**Table 1:** Basic characteristics of included studies.

Author	Year	Country	Sample size	Pathological type	Treatment	Tumor stage	Cutoff value of AAPR	Survival endpoint	NOS
Li^14^	2019	China	290	NSCLC	Non-surgery	TNM IV	0.36	OS	8
Li^15^	2019	China	390	NSCLC	Surgery	TNM I-IIIA	0.57	OS, PFS	8
Li^16^	2019	China	122	SCLC	Non-surgery	Limited stage	0.61	OS, PFS	7
Zhang^17^	2019	China	496	NSCLC	Surgery	TNM I-III	0.64	OS, PFS	8
Li^18^	2020	China	300	SCLC	Non-surgery	Extensive stage	0.52	OS, PFS	7
Zhou^19^	2020	China	808	NSCLC	Non-surgery	TNM III-IV	0.34	OS	7
Zhou^20^	2020	China	224	SCLC	Non-surgery	Extensive stage	0.35	OS	8
Birgitte^21^	2021	Denmark	5979	NSCLC	Mixed	TNM I-IV	0.35	OS	7
Birgitte^21^	2021	Denmark	1099	SCLC	Mixed	TNM I-IV	0.35	OS	7
Liu^22^	2021	China	167	NSCLC	Non-surgery	Advanced	0.238	OS, PFS	8
Gan^23^	2022	China	598	NSCLC	Non-surgery	TNM IIIB-IV	0.47	OS, PFS	7
Hu^24^	2022	China	116	NSCLC	Mixed	TNM I-IV	0.35	OS	6

NSCLC, non-small cell lung cancer; SCLC, small cell lung cancer; TNM, tumor-node-metastasis; AAPR, albumin-to-alkaline phosphatase ratio; OS, overall survival; PFS, progression-free survival; NOS, NOS: Newcastle–Ottawa Scale.

### The association between pretreatment AAPR and OS

**Figure 2:**
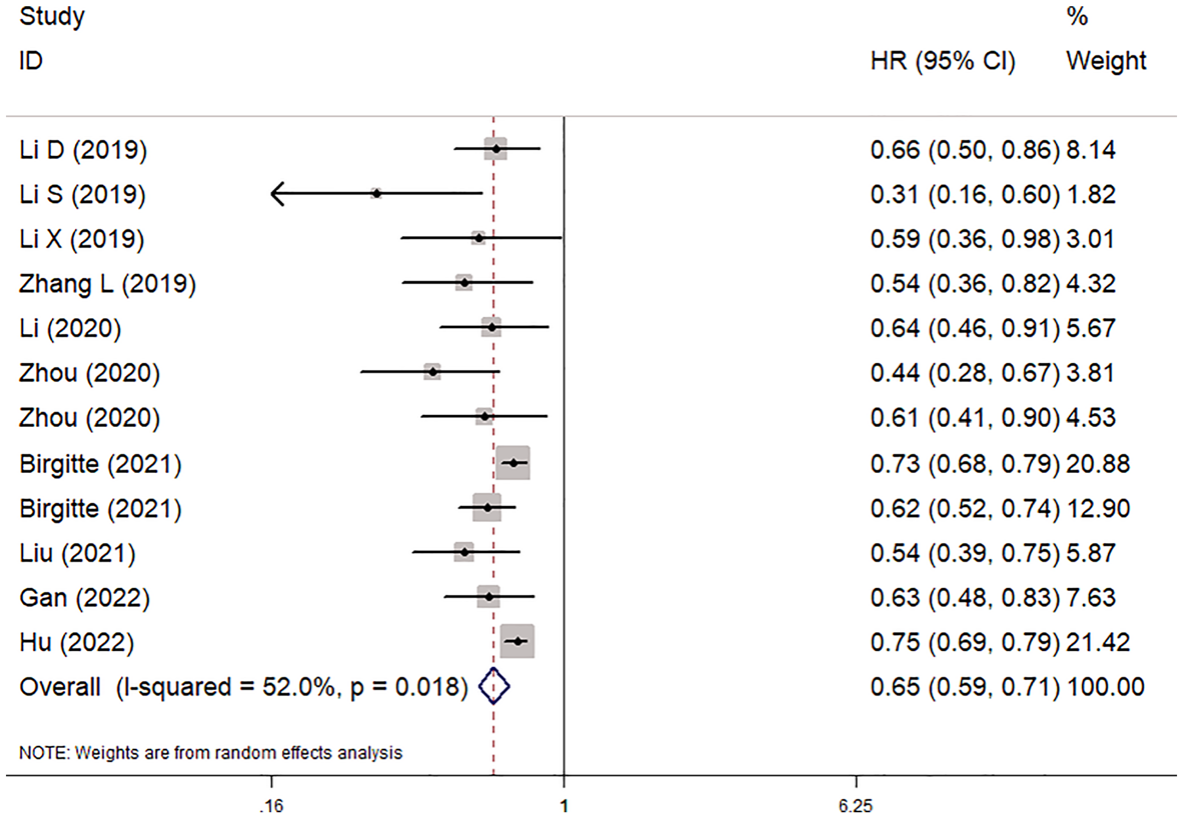
The association between pretreatment albumin-to-alkaline phosphatase ratio and overall survival of lung cancer patients.

All 11 included studies explored the predictive role of pretreatment AAPR for OS of lung cancer^14–24^. The pooled results demonstrated that a lower pretreatment AAPR was significantly associated with poorer OS (HR = 0.65, 95% CI 0.59–0.71, *P* < 0.001; I^2^ = 52.0%, *P* = 0.018) (Fig. 2). Then, subgroup analysis based on the pathological type (NSCLC vs SCLC) and treatment (non-surgery vs. surgery vs. mixed) also showed that a lower pretreatment AAPR was related to worse OS (NSCLC: HR = 0.65, 95% CI 0.58–0.73, *P* < 0.001; SCLC: HR = 0.62, 95% CI 0.54–0.71, *P* < 0.001; non-surgery: HR = 0.60, 95% CI 0.53–0.68, *P* < 0.001; surgery: HR = 0.46, 95% CI 0.33–0.65, *P* < 0.001; mixed: HR = 0.65, 95% CI 0.59–0.71, *P* < 0.001) (Table 2).

**Table 2:** The association between albumin-to-alkaline phosphatase ratio and prognosis in lung cancer.

	No. of studies	Hazard ratio	95% confidence interval	P value	I^2^ (%)	P value
Overall survival	11^14–24^	0.65	0.59–0.71	< 0.001	52.0	0.018
Pathological type						
NSCLC	8^14,15,17,19,21–24^	0.65	0.58–0.73	< 0.001	63.0	0.008
SCLC	4^16,18,20,21^	0.62	0.54–0.71	< 0.001	0.0	0.994
Treatment						
Non-surgery	7^14,16,18–20,22,23^	0.60	0.53–0.68	< 0.001	0.0	0.804
Surgery	2^15,17^	0.46	0.33–0.65	< 0.001	47.8	0.166
Mixed	2^21,24^	0.73	0.70–0.77	< 0.001	48.7	0.142
Progression-free survival	6^15–18,22,23^	0.68	0.59–0.78	< 0.001	32.5	0.192
Pathological type						
NSCLC	4^15,17,22,23^	0.70	0.60–0.80	< 0.001	54.0	0.089
SCLC	2^16,18^	0.59	0.40–0.86	0.007	0.0	0.632
Treatment						
Non-surgery	4^16,18,22,23^	0.72	0.62–0.84	< 0.001	36.7	0.192
Surgery	2^15,17^	0.55	0.40–0.74	< 0.001	0.0	0.603

NSCLC, non-small cell lung cancer; SCLC, small cell lung cancer.

### The association between pretreatment AAPR and PFS

**Figure 3:**
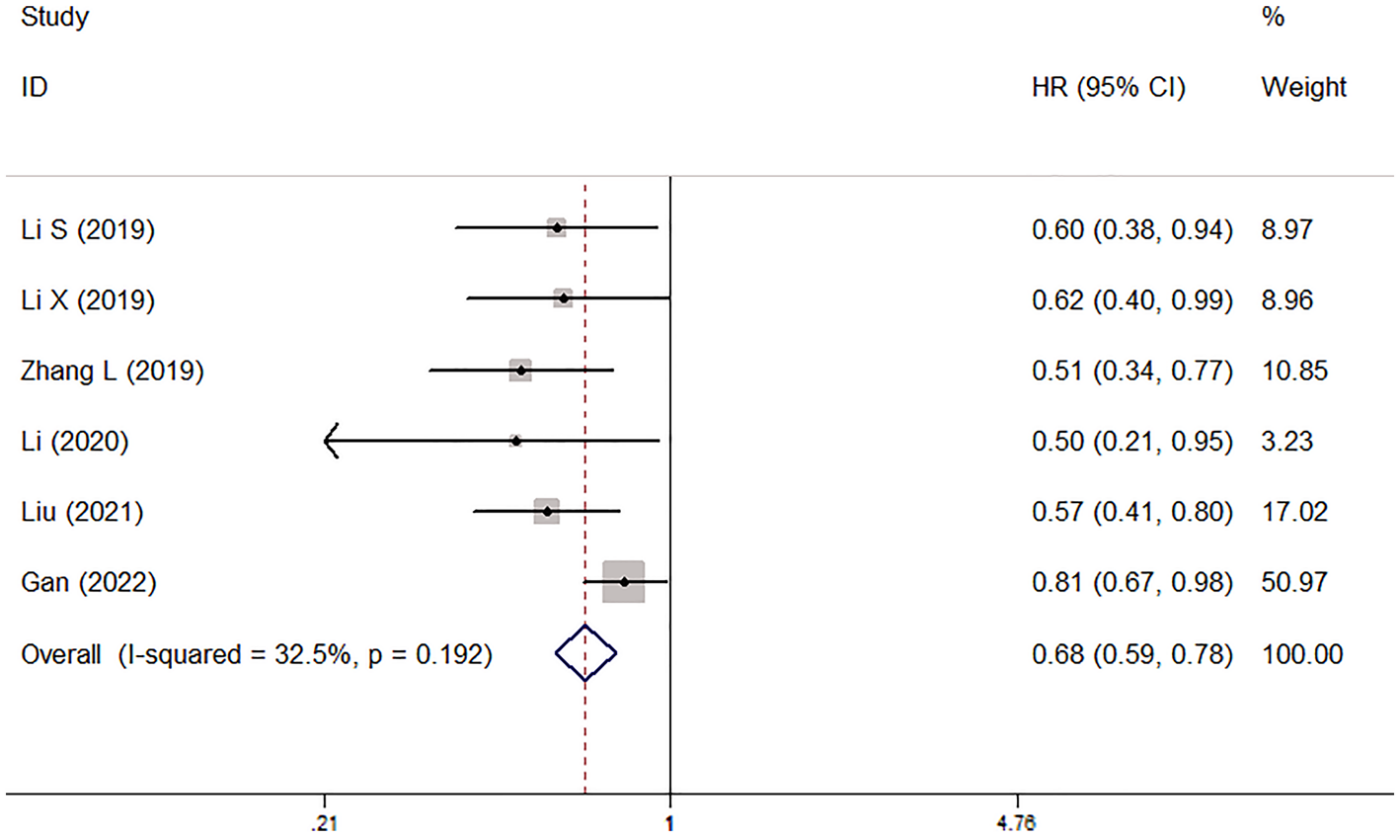
The association between pretreatment albumin-to-alkaline phosphatase ratio and progression-free survival of lung cancer patients.

Six studies explored the association between pretreatment AAPR and PFS of lung cancer patients^15–18,22,23^. The pooled results indicated that pretreatment AAPR was obviously related to poor PFS (HR = 0.68, 95% CI 0.59–0.78, *P* < 0.001; I^2^ = 32.5%, *P* = 0.192) (Fig. 3). Subgroup analysis stratified by the pathological type and treatment manifested similar results (NSCLC: HR = 0.70, 95% CI 0.60–0.80, *P* < 0.001; SCLC: HR = 0.59, 95% CI 0.40–0.86, *P* = 0.007; non-surgery: HR = 0.72, 95% CI 0.62–0.84, *P* < 0.001; surgery: HR = 0.55, 95% CI 0.40–0.74, *P* < 0.001) (Table 2).

### The association between pretreatment AAPR and clinicopathological parameters

Based on available data, we identified the relationship between pretreatment AAPR and age, gender, smoking history, differentiation degree, T stage, N stage and TNM stage. Pooled results indicated that lower pretreatment AAPR was significantly associated with male (RR = 1.08, 95% CI 1.03–1.13, *P* < 0.001; I^2^ = 34.3%, *P* = 0.124), poor differentiation (RR = 1.33, 95% CI 1.03–1.73, *P* = 0.029; I^2^ = 0.0%, *P* = 0.380), advanced T stage (RR = 1.25, 95% CI 1.03–1.52, *P* = 0.026; I^2^ = 59.6%, *P* = 0.060), N stage (RR = 1.34, 95% CI 1.15–1.55, *P* < 0.001; I^2^ = 0.0%, *P* = 0.610) and TNM stage (RR = 1.14, 95% CI 1.06–1.223, *P* < 0.001; I^2^ = 87.9%, *P* < 0.001) (Table 3).

**Table 3:** The association between albumin-to-alkaline phosphatase ratio and clinicopathological parameters in lung cancer.

Author	Age (older)	Gender (male)	Smoking history	Differentiation degree (low)	T stage (advanced)	N stage (advanced)	TNM stage (advanced)
Li^14^	1.027 (0.777–1.356)	1.054 (0.867–1.282)	1.012 (0.781–1.311)	–	–	–	–
Li^15^	1.096 (0.868–1.384)	1.050 (0.898–1.227)	1.206 (0.978–1.486)	1.175 (0.800–1.728)	1.225 (1.012–1.483)	1.746 (1.132–2.694)	2.029 (1.454–2.832)
Li^16^	–	–	–	–	–	–	–
Zhang^17^	1.145 (0.941–1.394)	1.013 (0.893–1.148)	1.115 (0.979–1.270)	–	1.864 (1.298–2.676)	1.330 (1.063–1.665)	1.450 (1.135–1.852)
Li^18^	1.000 (0.750–1.332)	1.089 (0.930–1.276)	1.028 (0.835–1.266)	–	1.190 (0.922–1.534)	1.250 (0.912–1.715)	–
Zhou^19^	0.755 (0.587–0.971)	1.083 (0.986–1.191)	1.085 (0.945–1.246)	–	–	–	1.134 (1.076–1.195)
Zhou^20^	1.075 (0.825–1.402)	1.127 (1.039–1.223)	1.042 (0.906–1.197)	–	–	–	–
Birgitte^21^	–	1.064 (1.012–1.119)	1.001 (0.986–1.017)	–	–	–	1.177 (1.139–1.216)
Birgitte^21^	–	1.276 (1.134–1.437)	–	–	–	–	1.058 (1.023–1.093)
Liu^22^	0.982 (0.780–1.236)	0.821 (0.630–1.069)	–	1.484 (1.045–2.109)	1.031 (0.822–1.294)	1.243 (0.896–1.725)	0.957 (0.762–1.201)
Gan^23^	1.094 (0.921–1.298)	1.115 (0.925–1.344)	1.315 (0.991–1.745)	–	–	–	1.024 (0.987–1.062)
Hu^24^	–	0.854 (0.514–1.419)	0.980 (0.781–1.230)	–	–	–	1.207 (0.969–1.504)
Poole RR with 95% CI	1.03 (0.95–1.12), * P* = 0.448	1.08 (1.03–1.13), * P* < 0.001	1.00 (0.99–1.02), * P* = 0.537	1.33 (1.03–1.73), * P* = 0.029	1.25 (1.03–1.52), * P* = 0.026	1.34 (1.15–1.55), * P* < 0.001	1.14 (1.06–1.22), * P* < 0.001

RR, relative risk; CI, confidence interval.

### Sensitivity analysis and publication bias

**Figure 4:**
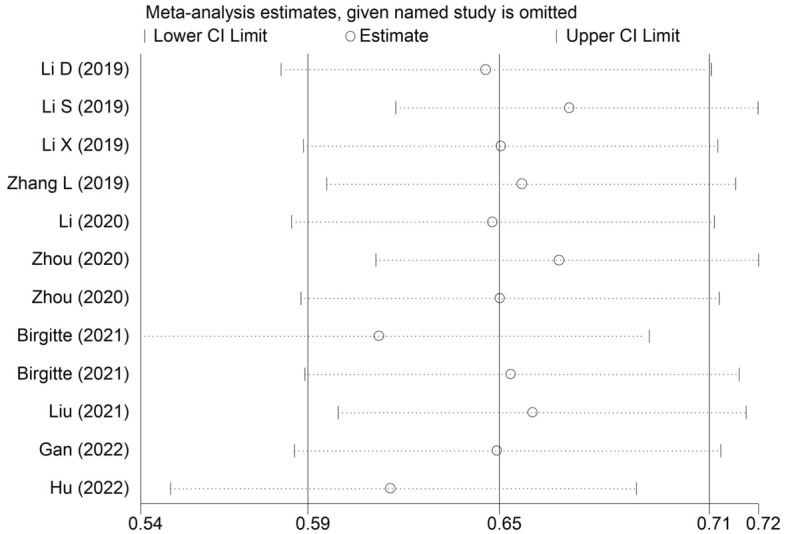
Sensitivity analysis about the association between pretreatment albumin-to-alkaline phosphatase ratio and overall survival of lung cancer patients.

**Figure 5:**
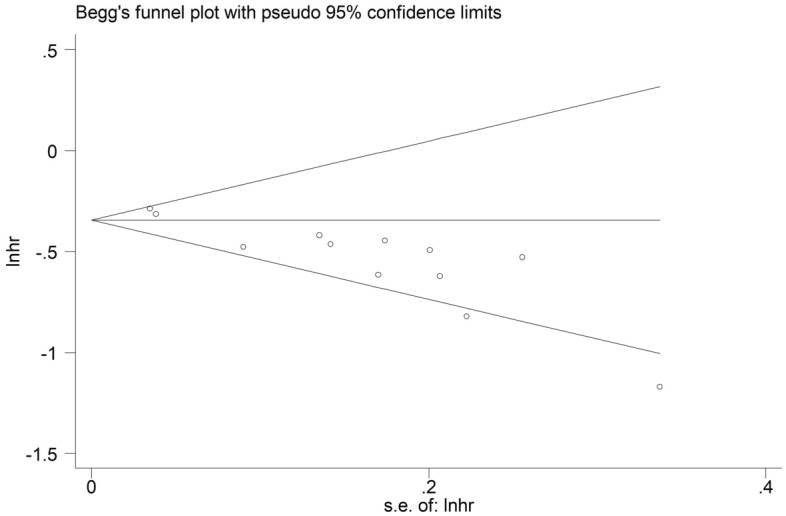
Begg’s funnel plot for the association between pretreatment albumin-to-alkaline phosphatase ratio and overall survival of lung cancer patients.

**Figure 6:**
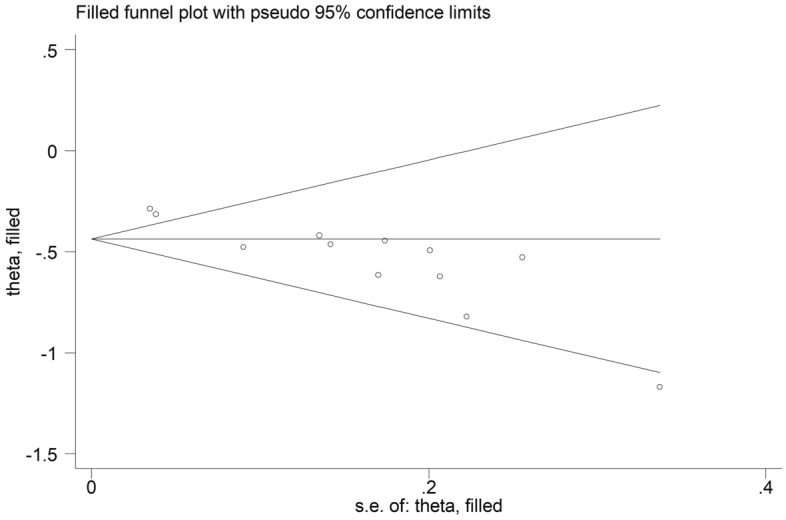
Filled Begg’s funnel plot for the association between pretreatment albumin-to-alkaline phosphatase ratio and overall survival of lung cancer patients.

The sensitivity analysis for the OS showed that our results were stable and reliable (Fig. 4). Due to the asymmetrical Begg’s funnel plot (Fig. 5) and *P* < 0.001 of Egger’s test, obvious publication bias was detected. Therefore, the nonparametric trim-and-fill method was used to identify potentially unpublished studies. However, no potentially unpublished studies were detected (Fig. 6). Thus, more high-quality studies are still needed to verify our findings.

## Discussion

The current meta-analysis demonstrated that pretreatment AAPR was significantly associated with prognosis and tumor stage and lung cancer patients with a lower pretreatment AAPR experienced poorer survival and advanced tumor stage. According to our findings, the pretreatment AAPR is a novel and reliable prognostic factor in lung cancer. However, more prospective high-quality studies are still needed to verify our findings due to limitations existed in this meta-analysis.

AAPR, as a novel parameter which has been reported in the past few years, is defined as the ratio of the serum albumin and serum alkaline phosphatase. It has been well manifested that the albumin level is related to the development, progression and prognosis among cancer patients^25–27^. Albumin concentration could reflect the nutritional status, liver function and human defense capabilities^23^. Decreased albumin concentration indicates the sign of malnutrition and decrease in immunity and the production of albumin is suppressed by the activation of inflammatory cytokines^28^. Besides, it has been also reported that the alteration in protein binding shows an impact on the drug half-life, which means hypoalbuminemia could cause a poor response to anti-tumor therapeutics^29^. Therefore, albumin is closely related to the disease progression and prognosis of cancers^30–32^. On the other hand, alkaline phosphatase is a novel factor predicting the survival of cancer patients. It is a hydrolase enzyme that widely exists in the kidney, liver and bone. Thus, alkaline phosphatase is usually applied as a biomarker reflecting the liver and bone health. In recent years, alkaline phosphatase has been demonstrated to play an important role in the anti-inflammation and immune response^33^. Meanwhile, numerous studies indicate that alkaline phosphatase concentration is also significantly related to the development and disease progression in cancers^34,35^. Besides, alkaline phosphatase has also been reported to associated with the survival in several types of cancers including the lung cancer^36^.

Although the association of serum and alkaline phosphatase concentration with prognosis in tumor patients have been reported^29–32,36^. Some studies suggest that AAPR is more effective in predicting the survival and prognosis of lung cancer patients compared to individual biomarkers^15–18,22,23^. It demonstrates greater sensitivity in capturing the patient’s physical condition and disease progression, thus holding stronger predictive value^15–18,22,23^. Moreover, monitoring changes in AAPR during the course of treatment allows for a more comprehensive understanding of the patient’s response to therapy. The effectiveness of treatment may manifest in various aspects, including cellular activity, inflammation levels, and protein metabolism, and AAPR, as a composite indicator, is better positioned to reflect these complex biological changes. Therefore, AAPR is considered to show greater clinical value than the individual levels of albumin and alkaline phosphatase.

Actually, the prognosis role of pretreatment AAPR has been verified in some cancers^37,38^. An et al. conducted a meta-analysis by including 18 studies involving 25 cohorts with 7019 cancer patients and revealed that decreased AAPR was significantly related to poor OS (HR = 2.14, 95% CI 1.83–2.51), disease-free survival (DFS) (HR = 1.81, 95% CI 1.60–2.04), PFS (HR = 1.71, 95% CI 1.31–2.22) and cancer-specific survival (CSS) (HR = 2.22, 95% CI 1.67–2.95)^37^. Notably, in their meta-analysis only five included studies focused on lung cancer^37^. Besides, Zhang et al. included 12 cohorts and demonstrated that lower AAPR predicted significantly poorer OS (HR = 2.02, 95% CI 1.78–2.30) and RFS (HR = 1.88, 95% CI 1.37–2.57) in hepatocellular carcinoma patients^37^. This was the first meta-analysis to identify the prognosis role of AAPR in lung cancer and our results strongly indicated the association of lower pretreatment AAPR with worse survival of lung cancer patients.

According to current evidence, we speculate some application of AAPR in clinics. AAPR is used as a prognostic indicator in assessing lung cancer patients. A lower AAPR is typically associated with an unfavorable prognosis, indicating a poorer physical condition in patients and a higher susceptibility to malignant tumor-related complications. Physicians can monitor changes in AAPR before and after treatment to assess the effectiveness of therapy and the overall condition of the patient. AAPR can serve as an auxiliary metric, aiding doctors in devising more personalized treatment plans. For patients with a lower AAPR, more proactive supportive therapies, such as nutritional support or adjustments to the treatment plan, may be necessary to enhance treatment tolerance. Changes in AAPR can also be utilized to monitor disease recurrence in lung cancer patients. Following treatment, a rapid decline in AAPR may suggest tumor recurrence or progression, prompting physicians to undertake further examinations or adjust the treatment plan. However, AAPR should be considered as part of a comprehensive evaluation alongside other clinical and laboratory indicators, rather than being used in isolation. Other factors influencing AAPR, such as infections, inflammation, and other chronic diseases, should also be taken into account. Additionally, caution is advised when interpreting AAPR results, as they may be influenced by various factors. Clinical practitioners should integrate AAPR with the overall patient profile and other relevant indicators for a more accurate assessment of the patient’s disease status and the formulation of individualized treatment plans.

Several limitations existed in our meta-analysis. First, all included studies are retrospective with relatively small sample sizes. Second, most included studies are from China, limiting the generalizability of our results. Third, it was unable to conduct more subgroup analysis based on other important parameters such as the tumor stage and age. Four, it is not feasible to determine the optimal threshold of AAPR due to the lack of original data.

## Conclusion

Pretreatment AAPR is significantly related to prognosis and tumor stage in lung cancer and patients with a lower pretreatment AAPR are more likely to experience poor survival and advanced tumor stage. Therefore, the pretreatment AAPR could serve as a novel and valuable prognostic factor in lung cancer patients.

## Data Availability

All data generated or analyzed during this study are included in this published article.
